# The global governance of antimicrobial resistance: a cross-country study of alignment between the global action plan and national action plans

**DOI:** 10.1186/s12992-020-00639-3

**Published:** 2020-11-11

**Authors:** Louise Munkholm, Olivier Rubin

**Affiliations:** Department of Social Sciences and Businesses, Roskilde University, Universitetsvej 1, 4000 Roskilde, Denmark

**Keywords:** Antimicrobial resistance, National action plans, Policy alignment, Comparative research

## Abstract

**Background:**

Antimicrobial resistance (AMR) is a growing problem worldwide in need of global coordinated action. With the endorsement of the Global Action Plan (GAP) on AMR in 2015, the 194 member states of the World Health Organization committed to integrating the five objectives and corresponding actions of the GAP into national action plans (NAPs) on AMR. The article analyzes patterns of alignment between existing NAPs and the GAP, bringing to the fore new methodologies for exploring the relationship between globally driven health policies and activities at the national level, taking income, geography and governance factors into account.

**Methods:**

The article investigates the global governance of AMR. Concretely, two proxies are devised to measure vertical and horizontal alignment between the GAP and existing NAPs: (i) a syntactic indicator measuring the degree of verbatim overlap between the GAP and the NAPs; and (ii) a content indicator measuring the extent to which the objectives and corresponding actions outlined in the GAP are addressed in the NAPs. Vertical alignment is measured by the extent to which each NAP overlaps with the GAP. Horizontal alignment is explored by measuring the degree to which NAPs overlap with other NAPs across regions and income groups. In addition, NAP implementation is explored using the Global Database for Antimicrobial Resistance Country Self-Assessment.

**Findings:**

We find strong evidence of vertical alignment, particularly among low-income countries and lower-middle-income countries but weaker evidence of horizontal alignment within regions. In general, we find the NAPs in our sample to be mostly aligned with the GAP’s five overarching objectives while only moderately aligned with the recommended corresponding actions. Furthermore, we see several cases of what can be termed ‘isomorphic mimicry’, characterized by strong alignment in the policies outlined but much lower levels of alignment in terms of actual implemented policies.

**Conclusion:**

To strengthen the alignment of national AMR policies, we recommend global governance initiatives based on individualized responsibilities some of which should be legally binding. Our study provides limited evidence of horizontal alignment within regions, which implies that regional governance institutions (e.g., WHO regional offices) should primarily act as mediators between global and local demands to strengthen a global governance regime that minimizes policy fragmentation and mimicry behavior across member states.

**Supplementary Information:**

The online version contains supplementary material available at 10.1186/s12992-020-00639-3.

## Background

Antimicrobial resistance (AMR) constitutes a complex and transboundary global health crisis estimated to be responsible for 700,000 deaths worldwide annually, causing healthcare systems excess costs in the tens of billions of US dollars [1–3]. Coordinated global stewardship is pertinent to effectively mitigate the threat [4–9]. In September 2013, the World Health Organization (WHO) Strategic Technical Advisory Group on AMR recommended developing a Global Action Plan (GAP) on AMR. The recommendation was subsequently adopted in May 2014 as a World Health Assembly resolution, and WHO started drafting the GAP together with the Food and Agriculture Organization of the United Nations (FAO) and the World Organization for Animal Health (OIE) (henceforth ‘the Tripartite’). The GAP was endorsed in May 2015 by the WHO’s 194 member states who were urged to develop and have in place national action plans (NAPs) on AMR by 2017 modeled on the guidelines in the GAP.

An important task for the Tripartite is to push forward national progress on the harmonization of AMR policies. The article aims to inform this task by analyzing patterns of alignment between existing NAPs and the GAP. It brings to the fore new methodologies for exploring the relationship between globally driven health policies and activities at the national level, taking income, geography and governance factors into account. Thereby, the article contributes to the global health governance literature by illuminating the important juncture between governance initiatives at the global level and then alignment at the national level.

The GAP promotes five strategic objectives: (i) improve awareness and understanding of antimicrobial resistance through effective communication, education, and training; (ii) strengthen the knowledge and evidence base through surveillance and research; (iii) reduce the incidence of infection through effective sanitation, hygiene and infection prevention measures; (iv) optimize the use of antimicrobial medicines in human and animal health; and (v) develop the economic case for sustainable investment that takes account of the needs of all countries, and increase investment in new medicines, diagnostic tools, vaccines, and other interventions [10].

To monitor national progress, the Tripartite relies on self-reporting by member states on AMR-related policies made available in the Global Database for Antimicrobial Resistance Country Self-Assessment [11]. The database is based on surveys submitted annually to all member states. Currently, the database contains three separate surveys covering the periods 2016–2017, 2017–2018, and 2018–2019. The database is unique in terms of its level of detail (with around hundred questions pertaining to different dimensions of AMR stewardship) and outreach (around 90% of member states have participated in the surveys). There is evidence that such self-reported assessments in global health might be able to accurately reflect real health initiatives and capacities across different countries, in particular if the assessment is combined with regular external evaluations [12]. In addition, self-assessments can also serve as an effective tool for maintaining momentum and commitment to the global policy process.

However, as a tool for accurate and objective information on these initiatives, the Global Database for Antimicrobial Resistance Country Self-Assessment suffers from some methodological deficiencies. First, self-reporting is based on subjective interpretations and scorings with limited opportunities for third-party triangulation and validation of the reported scores. Second, this in turn, introduces some arbitrariness as much depends on the person(s) filling out the survey, which would explain counterintuitive responses over time. Latvia, for example, goes from reportedly having approved their NAP in parliament in 2018 to merely having it under development a year later in 2019. Thirdly, the dataset appears to include some inconsistencies that could be ascribed mistakes in the manual reporting such as when Cambodia suddenly reports the highest value for NAP implementation in 2018, despite the fact that no data was made available that year. Fourth, some questions are not similar from one survey to the next, which impedes the scope for diachronic analyses. Lastly, the questions are not equipped to capture alignment along all the five stated objectives of the GAP. While questions pertaining to AMR awareness and surveillance (objectives one and two) are amply represented in the survey, alignment with objectives pertaining to infection prevention and control, optimal usage of medicines, and sustainable investments in new diagnostic tools (objectives three, four and five) are much harder to ascertain.

At this point, however, no other comparable global datasets on AMR policy alignment are available. The WHO is working on a richer dataset on AMR policies but that is projected to take five to ten years to compile [13]. Today, the WHO initiative of establishing the Global Antimicrobial Resistance Surveillance System (GLASS) comes closest to such dataset but currently only 87 member states participate to varying degree, and the reporting system is also based on self-reporting [14]. Two studies have addressed AMR policy alignment across several NAPs within specific regions [15, 16]. However, these studies do not make use of systematic indicators that can cut across member states, and their focus on only a few regions does not allow for comparison across regions.

In the absence of objective methods for measuring NAP alignment with the GAP, this article develops two indicators that can be used as proxies for AMR policy alignment both vertically and horizontally. The syntactic indicator measures the degree of verbatim overlap between the NAPs and the GAP while the second content indicator measures the degree to which the NAPs align with the five objectives and the corresponding actions in the GAP. A key aim of the article is to probe the extent to which the NAPs are driven by the Tripartite’s GAP (vertical alignment), and to what extent they are driven by regional and/or income dynamics (horizontal alignment). These indicators are triangulated with the existing subjective data from the Global Database for Antimicrobial Resistance Country Self-Assessment to provide a more comprehensive picture of progress in global AMR stewardship.

## Methods

The first indicator measures the syntactic overlaps between the NAPs and the GAP (vertical alignment) and between the different NAPs (horizontal alignment). We used plagiarism software to construct this indicator that provides information on the extent to which individual NAPs overlap verbatim with each other or the GAP (Plagiarism Checker X, version 6.0.9). The indicator measures alignment in terms of vocabulary and syntactic overlap and thus captures the extent to which NAPs make use of identical discourse vertically with the GAP and horizontally with other NAPs. However, the indicator does not provide a reliable measure of the degree to which member states actually specify and commit to the five key objectives and corresponding policy initiatives outlined in the GAP. To capture this important dimension a more complex content analysis is merited.

The second indicator, therefore, relies on a systematic content analysis of the NAPs using NVivo software (NVivo Pro, version 12). The indicator focuses on the extent to which the five strategic objectives of the GAP have been addressed in the NAPs. Under each objective, a number of so-called 'corresponding actions' to be taken by the member states are laid out [10]. We developed a codebook based on the GAP in which we turned objectives and corresponding actions into Text Search Query Items (TSQIs) that were then used to code the content of all NAPs using the NVivo text search query tool (see Additional file 1: Codebook for NVivo content analysis). Concretely, the five objectives were turned into 12 TSQIs based on key words, such as ‘awareness’, ‘surveillance’ and ‘hygiene’ to proxy for alignment with the objectives. The same procedure was applied to the corresponding actions which were turned into 39 TSQIs based on the overarching criteria that they were formulated as a specific demand from WHO. For example, the GAP demands ‘participation in an annual world antibiotic awareness campaign’ under objective one (TSQI ‘world antibiotic awareness’) and ‘implementation of the recommendations of the WHO Advisory Group on Integrated Surveillance of Antimicrobial Resistance’ under objective two (TSQI ‘WHO Advisory Group on Integrated Surveillance of Antimicrobial Resistance’ and AGISAR) [10]. The TSQIs allowed us to score all NAPs by their alignment to the GAP in terms of the objectives and corresponding actions. Importantly, the score on corresponding actions can be disaggregated according to each of the five objectives, which provides data on of the relative importance placed on each objective in the NAPs.

Vertical and horizontal alignment is explored by disaggregating the syntactic and content indicators according to geography and income. This allows us to identify regional patterns (the extent to which countries in the same region show similar degrees of alignment) and patterns related to income groups (the extent to which alignment is determined by income). The syntactic indicator provides a measure of overlap in percentages while the content indicator proxies alignment with the GAP objectives and corresponding actions by providing a score between 0 (no alignment) and 51 (perfect alignment). The study applies the regional categories of the WHO: the African Region (AFRO), the South-East Asia Region (SEARO), the Western Pacific Region (WPRO), the Region of the Americas (PAHO), the Eastern Mediterranean Region (EMRO), and the European Region (EURO) [17]. World Bank categories are applied to categorize income groups: low-income countries (LICs), lower-middle-income countries (LMICs), upper-middle-income countries (UMICs) and high-income countries (HICs) [18]. Data on income per capita (GNI per capita in 2018, Atlas method, current US$) is taken from the World Development Indicators [19]. All statistical tests in our study are based on 95% confidence levels/intervals, and the full dataset together with the key statistical outputs are available as additional files (see Additional file 2: Dataset syntax indicator and Additional file 3: Dataset content indicator).

**Fig. 1:**
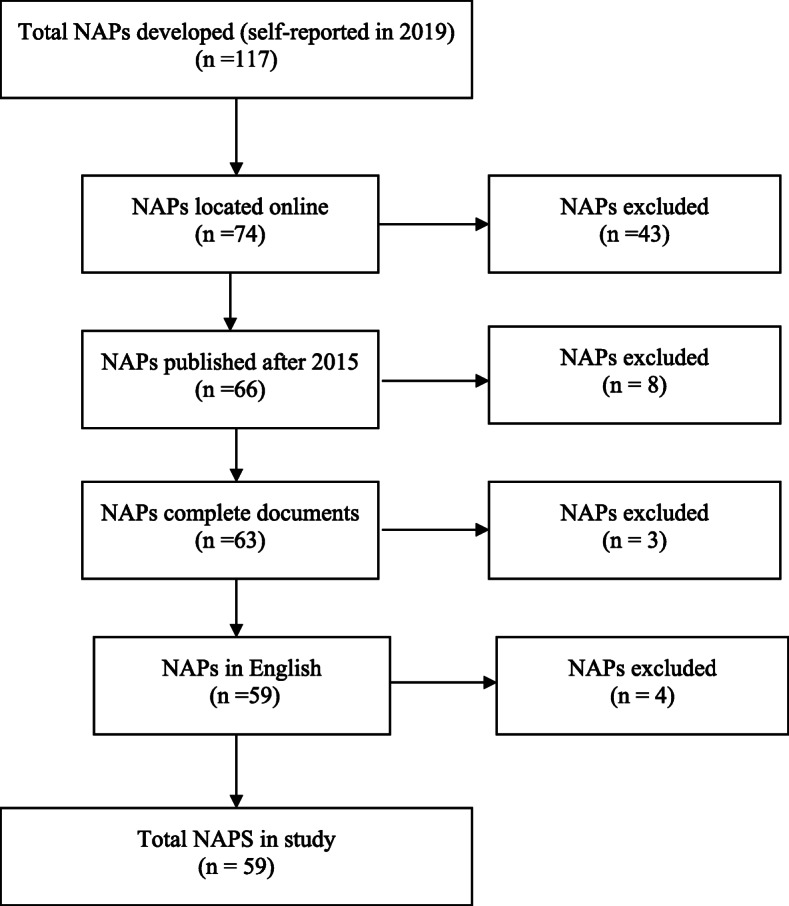
Sample process: Selection of NAPs for the study

In terms of sample, we included NAPs that could be found on the WHO website or through the latest self-reporting questionnaire to the Tripartite. In 2019, 117 member states reported to have developed NAPs [11]. However, only 74 NAPs have been published on the WHO website [20] or linked to in the self-reporting questionnaire [11]. The sample was purposely restricted to only include the NAPs developed after the publication of the GAP in 2015, because we were interested in assessing the vertical alignment with the GAP. This reduced the number of NAPs in our sample by eight to 66 NAPs. Only official national action plans in their full length were included (thus excluding brief letters to parliament, summaries or leaflets). This reduced our sample by three. We also had to restrict the sample to NAPs written in English in order to ensure consistency in the syntax and content analyses. This reduced our sample by four, resulting in a sample of 59 NAPs: 10 from AFRO, 11 from SEARO, nine from WPRO, three from PAHO, 11 from EMRO, and 15 from EURO. Figure 1 summarizes the selection process.

This limited sample can potentially weaken the generalizability of the results as the member states making their NAPs readily available online are not likely to constitute a randomly selected subsample of the existing NAPs. However, the bias is not likely to be substantial. The sampled NAPs exhibit the same regional diversity as the full population of NAPs, and there is no significant difference in mean income levels (t (112) = − 1.62; *p* = 0.11) between the member states that published their NAPs online (M = 20.01; SD = 23.77) and those that did not (M = 13.79; SD = 16.81). The potential bias, though, is likely to magnify when disaggregating data according to regions. In our sample, PAHO is only represented by three member states that have written their NAPs in English, thus excluding the NAPs written in Spanish from four Latin American member states. Thus, we err on the side of caution and classify this study as a plausibility probe [cf. [21]] that both provides a proof of concept in terms of useful techniques to investigate patterns of alignment *as well as* produces some robust results on overarching patterns of alignment, although these become less generalizable at more disaggregate levels.

## Results

This section presents our analytical findings regarding the current state of national AMR policy alignment, both vertically and horizontally, based on the syntax and content indicators as well as data regarding implementation status derived from the Global Database for Antimicrobial Resistance Country Self-Assessment.

### Vertical and horizontal alignment based on the syntactic indicator

The mean overlap between the GAP and the 59 NAPs is 8.4%. That is, on average more than 8% of the text in each NAP is similar (verbatim) to the GAP. Some NAPs overlap substantially more than the average such as Tanzania (14.6%), Indonesia (15.3%), and Kenya (16.1%). The Scandinavian NAPs are among the least overlapping documents with only 2.4% of the Danish NAP, 4.2% of the Swedish NAP and 4.7% of the Norwegian NAP being verbatim similar to the GAP. This tendency for developing (often African) countries to display high values on the syntactic indicator while developed (often European) countries have lower values suggests the existence of geography and income patterns. The bivariate correlation between income per capita and syntactic overlap shows a significant weak to medium negative effect of income on the extent of overlap: the poorer the country, the more it shares syntax with the international policy document driving the national alignment process (r = − 0.36; *n* = 56; *p* = 0.01).

Moving to horizontal alignment, we address whether distinct syntactic patterns can be attributed certain geographical regions. Syntactic patterns can manifest themselves in two ways. First, by the extent to which NAPs in a region overlap with other NAPs. This indicates the regional propensity for syntactic overlap with other NAPs in general and is calculated as the aggregated mean syntactic overlap of each NAP in the region with all other NAPs. Second, by the extent to which NAPs in a region overlap with other NAPs in the same region. This would indicate the propensity for syntactic overlaps with other NAPs within the same region and is calculated as the aggregated mean syntactic overlap of each NAP with other NAPs in the same region. Thus, the first horizontal set of data shows the regional pattern of verbatim overlap between NAPs (Table 1) while the second set of data shows the pattern of verbatim overlap within each region (Table 2).

**Table 1:** Regional pattern of syntactic overlap between NAPs

	Mean syntactic overlap (percentage)	Number of observations with syntactic overlap over 10%	Number of observations with syntactic overlap over 50%
SEARO	5.9	45	11
EMRO	5.5	56	1
AFRO	5.4	39	0
PAHO	4.4	4	0
EURO	3.9	16	0
WPRO	3.4	5	0

Legend: Sample of 59 NAPs; *n* = 3422 (each of the 59 NAPs are compared to the remaining 58). In descending order according to mean syntactic overlap

**Table 2:** Regional pattern of syntactic overlap between NAPs within the same region

	Mean syntactic overlap (percentage)	Number of observations with syntactic overlap over 10%	Number of observations with syntactic overlap over 50%
SEARO	10.8	14	10
EMRO	7.9	24	0
PAHO	7.0	1	0
AFRO	6.3	9	0
EURO	4.5	7	0
WPRO	3.7	1	0

Legend: Sample of 59 NAPs; *n* = 598 (each of the 59 NAPs are compared to other NAPs in the same region). In descending order according to mean syntactic overlap

Table 1 reveals regional patterns of syntactic overlap. SEARO displays the highest level of mean syntactic overlap (at 5.9%) followed by EMRO (5.5%) and AFRO (5.4%). The means of the three regions are significantly different from the lower mean values in EURO (3.9%) and WPRO (3.4%). Interestingly, the relatively high degree of syntactic overlap in EMRO appears to be driven by 56 observations with more than a 10% syntactic overlap between the NAPs, while the high mean in SEARO is driven by 11 extreme observations of more than 50% between the NAPs. Most notably, more than 50% of Afghanistan’s NAP is verbatim similar to India’s NAP, and 66% of India’s is similar to Afghanistan’s. Both NAPs are published around the same time in April/May 2017.

Investigating syntactic overlap *within* regions suggests an even greater variation in regional patterns (see Table 2). The mean overlap of the NAPs in SEARO is more than 10% compared to just 3.7 for the WPRO. The regional means in the two lowest scoring regions (EURO and WPRO) are significantly different from the two highest scoring regions (SEARO and EMRO). Again, the high level of syntactic overlap in SEARO is driven by many extreme values with overlaps of more than 50%. The NAPs from Indonesia, Timor-Leste, Maldives, and Myanmar, in particular, display high levels of syntactic overlap with each other, ranging between 50% and 65%.

We have already seen that vertical syntactic overlaps decrease by country income. Analyzing the extent of horizontal syntactic overlap in NAPs across income groups reveals a similar pattern. The group mean decreases as one moves towards higher income groups: LICs have a mean of 5.6%; LMICs have a mean of 5.4%; UMICs have a mean of 4.8% and HICs have a mean of 4.0%. The only income group mean that is significantly different from all the others, however, is the HICs.

In sum, it appears that the vertical alignment is the dominant dynamic in most member states. The mean of the vertical syntactic alignment is significantly higher at 8.4% compared to a mean of 4.7% for the horizontal regional alignment (t (58) = 18.3; *p* = 0.00). However, there is much more variation in the distribution of horizontal alignment with some NAPs, particularly from SEARO, overlapping with more than 60%.

### Vertical and horizontal alignment based on the content indicator

**Fig. 2:**
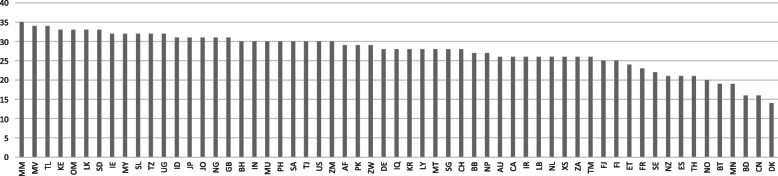
Total scores of NAPs based on TSQIs. Legend: Sample of 59 NAPs; *n* = 59. Scores reflect the total number of objectives and corresponding actions addressed in each NAP (represented by World Bank country code). Minimum score is 0 and maximum score is 51. In descending order according to scores (total)

Figure 2 illustrates the total scores of each NAP based on TSQIs measuring NAP alignment with the five GAP objectives (12 TSQIs) and their corresponding actions (39 TSQIs). The score varies from 14 (Denmark) to 35 (Myanmar) with a mean of 27.5. LMICs (including Myanmar, Timor-Leste, Kenya, Sri Lanka, Sudan, Indonesia, and Nigeria) and LICs (including Sierra Leone, Tanzania, and Uganda) dominate the top of the list whereas the least aligned member states are more economically diverse and include Denmark, China, Bangladesh, Mongolia, Bhutan, Norway, Thailand, Spain, and New Zealand. Geographically, member states belonging to AFRO, EMRO, and SEARO are overrepresented in the top half whereas countries belonging to the regions of EURO and WPRO dominate in the bottom half.

As with the syntactic indicator, the bivariate correlation between income per capita and the content indicator shows negative effect of income on the extent of alignment (the poorer the country, the more it shares content with the GAP), although the correlation is a bit weaker and only significant at a 10% level (r = − 0.24; *n* = 56; *p* = 0.073). This pattern, however, is not reproduced when looking at the differences across income groups: LICs have a mean of 28.8; LMICs have a mean of 29.2; UMICs have a mean of 26.8; and HICs have a mean of 26.4. None of the means are significantly different from each other.

Table 3 displays the regional patterns of content alignment. The table reveals a modest dispersion in content scores across regions with AFRO displaying the highest value of 29.9 and EURO the lowest of 25.3. None of the regional content scores are significantly different from each other, however.

**Table 3:** Regional patterns based on the content analysis

	Mean content overlap scores (total)	Mean content overlap scores (objectives)	Mean content overlap scores (actions)
AFRO	29.9	12.0	17.9
SEARO	29.4	11.8	17.5
WPRO	28.0	11.1	16.9
PAHO	27.7	11.3	16.3
EMRO	25.3	11.4	13.9
EURO	25.3	10.7	14.7

Legend: Sample of 59 NAPs; *n* = 59. Scores reflect the average number of objectives and corresponding actions included in NAPs belonging to the same region. Scores for mean content overlap (total): 0–51; scores for mean content overlap (objectives):0–12; scores for mean content overlap (actions): 0–39. In descending order according to mean content overlap (total)

In contrast to the syntactic indicator, the content indicator cannot capture horizontal alignment between NAPs within a region, as it is explicitly linked to alignment with the GAP. Instead, it can disaggregate data according to each of the five objectives. This provides information on the variation in alignment across objectives – something that was not possible with the syntactic indicator. Table 4 displays the degree of alignment under each of the five objectives and their related corresponding actions.

**Table 4:** Strategic objectives and actions from the GAP and their emphasis in the NAPs

	Percentage of general objective elements addressed in the average NAP	Percentage of corresponding actions addressed in the average NAP
Objective 1	97	31
Objective 2	100	39
Objective 3	87	76
Objective 4	100	35
Objective 5	95	43

Legend: Sample of 59 NAPs; *n* = 59

Table 4 suggests that most NAPs refer to the general elements of the five objectives whereas the level of alignment to the corresponding actions is much lower. The average NAP, for example, refers to 97% of the TSQIs under objective one (awareness, communic*, educat* and train*) while only addressing 31% of TSQIs of corresponding actions (e.g. ‘public communication programmes’, certif*, curricul* and ‘one health’). Thus, there is discrepancy between widespread referral to the five overarching objectives on the one side, and then the lack of specificity with regards to concrete actions on how to reach these objectives on the other. A noticeable exception is objective three that carries a high score with regards to both the objective (TSQIs of infect*, sanit* and hygiene) as well as corresponding actions (TSQIs such as ‘infection prevention’, ‘infection control’, susceptibility and vaccin*). Another corresponding action that seems to meet a high level of commitment with 50 out of 59 NAPs is the corresponding action of including ‘antimicrobial use and resistance in school curricula’ under objective one [10].

The corresponding actions that are mentioned the least are those referring to the implementation of central pieces of international law (e.g., only eight out of 59 refer to the International Health Regulations), international guidelines (e.g., only nine out of 59 refer to the recommendations of the WHO Advisory Group on Integrated Surveillance of AMR) and international codes for conduct (e.g., only three out of 59 refer to the OIE terrestrial and aquatic animal codes). Interestingly, the One Health approach is referred to in 55 NAPs but not all member states apply the approach when describing corresponding actions regarding how to coordinate efforts across sectors, incorporating humans, animals, plants and the broader environment; this is in accordance with the findings of other studies [15, 22]. Furthermore, attention to some corresponding actions appears to depend on income levels. All LICs and LMICs (except Mongolia, Bhutan and Bangladesh), for example, refer to ‘sanitation’ while many of the NAPs produced by UMICs and HICs do not engage with this issue in their NAPs. In comparison, HICs emphasize the proper use of ‘critical important antibiotics’ as well as ‘marketing authorization’ which are considered in only a couple of NAPs produced by LICs (the NAPs of Afghanistan and Tajikstan refers to critical important antibiotics, whereas none of the NAPs of LICs mention marketing authorization).

In sum, there is clearly a discrepancy between a high level of alignment with the five GAP objectives, and then a moderate alignment when it comes to the actual corresponding actions. Overall, the degree of vertical alignment with objectives and corresponding actions is higher in poorer member states, and it appears that attention to certain corresponding actions is dependent on income level. Significant regional differences could not be established based on the content indicator. Another key finding of the content analysis is the considerable attention across the NAPs given to the corresponding actions for objective 3, reducing the incidence of infection through effective infection prevention and control measures.

### Triangulating vertical and horizontal alignment with NAP implementation

Triangulating these alignment scores with the NAP implementation measure from the Global Database for Antimicrobial Resistance Country Self-Assessment produces some interesting findings. The implementation variable is measured on a five-point scale: 1) no national AMR action plan; 2) national AMR action plan under development or plan involves only one sector or ministry; 3) national AMR action plan developed that addresses human health, animal health, and other sectors; 4) multi-sectoral AMR action plan approved that reflects Global Action Plan objectives, with an operational plan and monitoring arrangements; and 5) multi-sectoral AMR action plan has funding sources identified, is being implemented and has monitoring in place [11]. Out of the 194 members of the WHO, 162 member states responded to the latest self-reporting survey (2018–2019). Of the 162 member states that responded to the latest survey, 117 reported to have developed NAPs (scoring three or above). However, only 26 member states report being in the process of implementing their NAPs (a score of five), which suggests very limited vertical alignment in the actual policies being implemented. Our subsample is predicated on the existence of a NAP, which excludes member states with an implementation score of one or two. Interestingly, we have not discerned any significant patterns in implementation scores across income or geography within our subsample. Table 5 illustrates very similar implementation score means across the different geographical regions. Not surprisingly, therefore, the means of syntactic and content overlap do not appear to correlate with the self-reported implementation scores. Thus, there is little evidence to suggest that having an elaborate NAP that is highly aligned with the GAP advances a smooth and fast process of implementation.

**Table 5:** Comparison of regional patterns in NAP implementation and content

Regions	Mean implementation scores (total)	Mean syntactic overlap (percentage)	Mean content overlap scores (total)
WPRO	4.6	3.7	28.0
AFRO	4.4	6.3	29.9
EURO	4.3	4.5	25.3
EMRO	4.1	7.9	25.3
PAHO	4.0	7.0	27.7
SEARO	4.0	10.8	29.4

Legend: Sample of 59 NAPs; *n* = 59. Mean implementation score (total): 3–5; mean syntactic overlap (percentage): 0–100; mean content overlap score: 0–51. In descending order according to mean implementation score (total)

## Discussion

Overall, the study finds evidence of vertical alignment in the AMR NAPs. The fact that 2/3 of member states, including some of the poorest member states, have developed NAPs within a very short period of time is a great achievement. As a point of comparison, the UN call for all member states in 2014 to develop NAPs to further the implementation of the UN Guiding Principles on Business and Human Rights (UNGPs) were only met by 21 countries four years into the call [23]. More than 95% of member states also report back to the Tripartite on national progress with regards to AMR policies. Notwithstanding this promising pattern of participation and alignment in the NAPs, the UN Interagency Coordination Group’s recent report to the UN Secretary General concludes that existing global AMR efforts ‘are currently too slow and must be accelerated’ [3]. As mentioned, only 26 member states report to be in the process of implementation, according to the latest survey [11]. In addition, only about half the NAPs that have been developed are made readily available online.

This juxtaposition between a high degree of vertical alignment in the policy documents and then much less harmonization and transparency in the actual policies implemented can be seen as a case of *isomorphic mimicry*. Isomorphic mimicry describes harmonization that takes place primarily in form and not in function [24–26]. Thus, policy documents and government procedures might give the appearance of having adopted best practices, but these practices are not implemented as intended [27]. The discrepancy between high alignment scores with respect to the more abstract objectives of the GAP and lower scores with regards to their concrete corresponding actions is also suggestive of isomorphic mimicry. Theory dictates that isomorphic mimicry is most often observed in LICs and LMICs in response to international guidelines [24]. These groups of countries are often faced with *capability traps* where governments might promise to undertake certain activities but subsequently fail to deliver due to a lack of capacity [24, 28, 29]. The urgency (a two-year timeframe) and scope (widespread participation by poorer member states) by which the NAPs have been developed increase the risks of capability traps or conflicting political concerns that could exacerbate mimicry behavior. Prior to 2015, very few member states had the equivalent of a NAP on AMR [30]. Also, most LICs and LMICs have many other (and sometimes conflicting) challenges that merit political attention [31]. A related conflicting health challenge, for example, is the lack of access to antibiotics, which by far eclipses AMR: almost six million deaths annually in LICs and LMICs can be attributed to the lack of access to antibiotics [6].

The UN Interagency Coordination Group concludes that the greatest challenge ‘is not writing a NAP but implementing it and demonstrating sustained action’ [32]. However, this study suggests that developing NAPs and implementing the corresponding actions should preferably come hand in hand. Global governance should not be perceived as two separate processes where the WHO designs universal policy guidelines and then leaves it to regional WHO offices and national governments to implement them. The fact that we see this disconnect between policy documents and actual practices indicates the need for addressing the policy process as a whole rather than reducing the challenge to one of policy implementation.

One way to strengthen global governance of AMR is to increase vertical alignment not just in terms of agenda conformity but also political commitment. There are several proponents for applying a ‘hard international law’ approach in the form of binding international treaties to push forward global concerted action in the AMR policy field [4–6, 8]. The argument is that an international legal intervention, such as a treaty, allows for more ambitious steps to be taken due to the introduction of binding obligations and sanction mechanisms for non-compliance. Establishing such a governance regime, however, is challenging and needs to address many dilemmas, such as distributional concerns, different national needs and demands, as well as the practical questions of resource allocation, capacity building, and how to establish an authority capable of enforcing such treaty. In comparison, legally binding regulations that target specific sectors and activities related to AMR would constitute a more pragmatic global governance regime. Such binding regulations could focus explicitly on reducing antimicrobials in livestock production, for example, by restricting the on-farm use of antimicrobials that are essentially important for human health [33]. Other GAP objectives might be less suitable for hard law solutions. Antimicrobial stewardship in health care services, for example, would likely have to be tailored carefully to the specific local contexts in which such activity is to be carried out, thus making it hard to define legally binding obligations at the global level. Therefore, it makes sense to build on the idea of ‘individualized responsibilities’ known from the Paris Climate Agreement where individualized targets and actions are determined at the national level but informed by a common global goal [8]. While at first glance this might seem similar to the current policy process on AMR based on NAPs, there are noticeable differences. First of all, the individualized responsibilities are legally binding. This means that countries commit to implement these nationally determined contributions within a confined period of time. At the same time, countries accept to be pledged, ratcheted, and reviewed every five years by an external, independent body that then measures levels of compliance. In addition, countries commit to annual meetings as well as global scientific appraisals every five years [8]. These governance tools are underdeveloped in the current monitoring of the GAP commitments that relies on self-reporting with limited possibility for third party verification. Thus, the introduction of individualized responsibilities that are legally binding represents a substantive change to the current global AMR governance regime. It could help reduce the propensity for isomorphic mimicry behavior as countries commit to undertake actions that are realistic seen from their perspective, which in turn might increase incentives to actually deliver. From a global governance perspective, making individualized responsibilities legally binding provides the Tripartite (or other global actors, such as the UN) with new tools for monitoring and enforcing global AMR initiatives.

It is vital that the NAP policy process does not merely lead to nationally ‘routinized responses in the form of tick-the-box activities that are designed to produce surface compliance’ [26]. The proposed recommendations – international treaties, international regulations, or international agreements with individualized responsibilities – have in common that they focus on strengthening the global AMR governance regime as a whole rather than focusing more narrowly on pushing forward national implementation of the GAP guidelines.

## Conclusion

In a relatively short time span, the Tripartite has succeeded in creating the basis for a global governance regime on AMR. More than 120 member states have now developed NAPs, and a monitoring regime based on self-reporting has been established. Our cross-country analysis of 59 NAPs contributes to an increased understanding of the variation in the content of the NAPs within and across regions and income groups. One key finding is that NAPs are more likely to align with the GAP in poorer member states, both when it comes to syntax (verbatim overlaps) and content (attention to objectives and corresponding actions). The NAPs in our sample are mostly aligned with the GAP’s five overarching objectives and only moderately aligned with the recommended corresponding actions. The study finds no relationship between NAP alignment with the GAP and reported implementation progress of the NAP. The study also finds limited evidence of strong horizontal alignment within regions. A few NAPs did exhibit substantial overlaps with each other where more than half their NAPs were identical, but, overall, the regional dynamics appear not be pronounced. Thus, strengthening the regional governance regime as a mediating level between global governance (the Tripartite and the GAP) and local delivery (national actors and NAPs), while preferable to uncoordinated national initiatives, will not solve the issue of limited global concerted action [34]. From a global governance perspective, horizontal alignment is most helpful when established across regions, across sectors and across stakeholders. Rather than regionalizing AMR governance, therefore, we argue for the need for strengthening global governance structures and approaches. The ideal role of WHO regional offices will be to support the development of the global governance regime, and to increase coordination across regions for implementing the GAP to avoid further fragmentation and to help minimizing mimicry behavior. Importantly, the global stewardship on AMR should not be reduced to a question of improving national implementation of GAP guidelines. Instead, this article argues in favor of strengthening the global policy process as a whole, not necessarily though ambitious and grandiose treaties but through international regulations based on legally binding individualized responsibilities.

## Supplementary Information

**Additional file 1.**

**Additional file 2.**

**Additional file 3.**

## Data Availability

All data generated and analyzed in the study have been included in this published article and its supplementary information files.
