# Supplementary material for: The global governance of antimicrobial resistance: a cross-country study of alignment between the global action plan and national action plans
Source: Global Health. 2020 Nov 11;16:109. doi: 10.1186/s12992-020-00639-3 (PMC7656753; doi:10.1186/s12992-020-00639-3)
Supplement: Supplementary file 1 — Additional file 1. [file 12992_2020_639_MOESM1_ESM.pdf]

Codebook for NVivo content analysis of NAPs

Procedure:

For our content analysis of the NAPs, we developed a codebook based on the GAP. The GAP promotes five overall strategic objectives and each of them is followed by a list of corresponding actions that member states are expected to respond to in their NAPs. We first produced an exhaustive list of policy initiatives demanded in the GAP relevant to the member states and used that list to identify text search query items (TSQIs). The TSQIs were then used to code the content of all NAPs using the NVivo text search query tool.

In our coding of the NAPs, we have strived to develop TSQIs in a way that enables us to obtain an overview of the extent to which NAPs align with the GAP. To avoid duplets in our coding, we have carefully worked out the codes on basis of a scrutiny of the GAP. For instance, when one action in the GAP entails one activity only, it will create one code in NVivo whereas when one action in the GAP entails more than one activity, the number of codes will reflect it.[1] Not all terms used in the GAP's description of its objectives and corresponding actions are easily turned into TSQIs and thus suitable for content analysis of the NAPs in NVivo due to their very broad and/or imprecise wording.[2]

The following presents the codebook.

[1] Examples: The corresponding action that reads 'participation in an annual world antibiotic awareness campaign' (objective 1) has been studied by the TSQI 'world antibiotic awareness' resulting in one code. In comparison, the action 'strengthen hygiene and infection prevention and control' (objective 3) has been studied using two TSQIs ('infection prevent*' and 'infection control') to reflect there here we have two different activities within one corresponding action, resulting in two codes.

[2] Examples: 'action across all government ministries' (objective 1), 'Consider implementing an agreed global public health research agenda on antimicrobial resistance' (objective 2), 'identification and elimination of economic incentives in all sectors that encourage inappropriate use of antimicrobial agents' (objective 4) and 'participate in international collaborative research to support the development of new medicines, diagnostic tools and vaccines' and 'piloting of innovative ideas for financing research' (objective 5).

Objective 1: Improve awareness and understanding of antimicrobial resistance through effective communication, education and training

Objective terms	TSQI	Corresponding actions	TSQI
Awareness	awareness	Increase national awareness of AMR through public communication programmes	"public communication programmes" + "public communication program"
Communication	communic*	Participation in an annual world antibiotic awareness campaign	"world antibiotic awareness"
Education	educat*	Establish AMR as a core component of professional education, training, certification and development	certif*
Training	train*	Include antimicrobial use and resistance in school curricula	curricul*
		Provide the public media with accurate and relevant information so that public information and reporting reinforce key messages	"public media"<br>"public information"
		Inclusion of AMR in national risk registers or other effective mechanisms for cross-government commitment	"risk register"<br>cross-govern*
		Establishment of multisectoral (one-health) coalitions to address AMR at local or national level	"one health"<br>coalition*

Objective 2: Strengthen the knowledge and evidence base through surveillance and research

Objective	TSQI	Corresponding actions	TSQI
Surveillance	surveil*	Develop a national surveillance system for AMR that:	"national surveillance system"
Research	research*	Includes a national reference centre	"national reference centre"
		Includes at least one reference laboratory	"reference laboratory"
		Strengthens surveillance in animal health and agriculture sectors by implementation of the recommendations of the WHO Advisory Group on Integrated Surveillance of AMR;	"WHO Advisory Group on Integrated Surveillance" + AGISAR
		the standards published in the OIE terrestrial and aquatic animal codes	"OIE terrestrial and aquatic animal codes" +"terrestrial code" + "Aquatic Animal Health Code"
		the FAO/WHO Codex Alimentarius Code of Practice to Minimize and Contain AMR	"Codex Alimentarius"
		the Codex Alimentarius Guidelines for Risk Analysis of Foodborne AMR	"Codex Alimentarius Guidelines for Risk Analysis" + "Guidelines for Risk Analysis" + "risk analysis"
		Has the capacity to detect and report newly emerged resistance that may constitute a public health emergency of international concern, as required under the International Health Regulations (2005)	"International Health Regulations"
		Collect and report data on use of antimicrobial agents in human and animal health and agriculture so that trends can be monitored	collect*<br>monitor*

Objective 3: Reduce the incidence of infection through effective sanitation, hygiene and infection prevention measures

Objective	TSQI	Corresponding actions	TSQI
Infection	infect*	Strengthen hygiene and infection prevention and control	"infection prevention"<br>"infection control"
Sanitation<br><br>Hygiene	sanit*<br><br>hygiene	Include within national surveillance of AMR the collection and reporting of data on antimicrobial susceptibility of microorganisms causing health care-associated infections	susceptibility<br>"health care associated"
		Implementation of the standards published in the OIE Terrestrial and Aquatic Animal Health Codes	"OIE terrestrial and aquatic animal codes" +"terrestrial code" + "Aquatic Animal Health Code"
		Implementation of FAO/WHO Codex Alimentarius Code of Practice to Minimize and Contain AMR	"Codex Alimentarius"
		Promote vaccination as a method of reducing infections in food animals	vaccin*

Objective 4: Optimize the use of antimicrobial medicines in human and animal health

Objective	TSQI	Corresponding actions	TSQI
Medicines	medicin*	Distribution, prescription, and dispensing of antimicrobials is carried out by accredited health or veterinary professionals under statutory body supervision	"accredited professionals"<br>"statutory body supervision"
		Marketing authorization is given only to antimicrobial agents that are quality assured	"marketing authorization" + "marketing authorisation"<br>assur*
		Development and implementation of national and institutional essential medicine lists guided by the WHO Model Lists of Essential Medicines	"model lists of essential medicines" + "model list of essential medicines" + "Essential Medicines List" + "Essential Medicines Lists" + EML
		Laboratory capacity to identify pathogens and their antimicrobial susceptibility in order to guide optimal use of medicines in clinical practice	"laboratory capacity"
		Provision of stewardship programmes	"stewardship programmes" + "stewardship program"
		Regulation and governance for licensing, distribution, use and quality assurance	licens*
		Implementation of Codex Alimentarius	"Codex Alimentarius"
		Implementation of WHO/OIE guidance on the use of critically important antibiotics	"critically important antibiotics"
		Phasing out of use of antibiotics for animal growth promotion	"phasing out"

Objective 5: Develop the economic case for sustainable investment that takes account of the needs of all countries, and increase investment in new medicines, diagnostic tools, vaccines and other interventions

Objective	TSQI	Corresponding actions	TSQI
Economic	econ*	Member States should consider assessing investment needs for implementation of their national actions plans on AMR, and should develop plans to secure and apply the required financing	finan*
Investment	invest*		
		Prioritization and support of basic scientific research on infectious diseases	“scientific research”
		Promote partnerships between research institutions in developed and developing countries	partner*
		Strengthening existing and creating new public-private partnerships for encouraging research and development of new antimicrobial agents and diagnostics	partner*
		Adoption of new market models to encourage investment and ensure access to new antimicrobial products	“market models” + “market model”
